# Added value of serial bio-adrenomedullin measurement in addition to lactate for the prognosis of septic patients admitted to ICU

**DOI:** 10.1186/s13054-020-2794-x

**Published:** 2020-02-28

**Authors:** Alice Blet, Charles de Roquetaillade, Oliver Hartmann, Joachim Struck, Alexandre Mebazaa, Benjamin Glenn Chousterman, on behalf of the Adrenoss-1 study investigators, Pierre-François Laterre, Caroline Berghe, Marie-France Dujardin, Suzanne Renard, Xavier Wittebole, Christine Collienne, Diego Castanares Zapatero, Thierry Dugernier, Marco Vinetti, Nicolas de Schryver, Anne Thirifays, Jacques Mairesse, Vincent Huberlant, Hélène Petre, Isabelle Buelens, Pierre Henin, Hugues Trine, Yves Laurent, Loix Sébastien, Paul Geukens, Laurent Kehl, Bruno François, Philippe Vignon, Nicolas Pichon, Emmanuelle Begot, Anne-Laure Fedou, Catherine Chapellas, Antoine Galy, Nicolas Rodier, Ludmilla Baudrillart, Michelle Nouaille, Séverine Laleu, Claire Mancia, Thomas Daix, Paul Bourzeix, Isabelle Herafa, Anne-Aurore Duchambon, Jean Baptiste Lascarrou, Maud Fiancette, Gwenhael Colin, Matthieu Henry-Lagarrigue, Jean-Claude Lacherade, Christine Lebert, Laurent Martin-Levfèvre, Isabelle Vinatier, Aihem Yehia, Konstantinos Bachoumas, Aurélie Joret, Jean Reignier, Cécille Rousseau, Natacha Maquigneau, Yolaine Alcourt, Vanessa Erragne Zinzonni, Angélique Deschamps, Angelina Robert, Emmanuelle Mercier, Véronique Simeon-Vieules, Aurélie Aubrey, Christine Mabilat, Denis Garot, Stephan Ehrmann, Annick Legras, Youenn Jouan, Pierre François Dequin, Antoine Guillon, Laetitia Bodet-Contentin, Emmannuelle Rouve, Charlotte Salmon, Lysiane Brick, Stéphanie Massat, Arnaud Desachy, Marie Anne Fally, Laurence Robin, Christophe Cracco, Charles Lafon, Sylvie Calvat, Stéphane Rouleau, David Schnell, Sigismond Lasocki, Philippe Fesard, Damien Leblanc, Guillaume Bouhours, Claire Chassier, Mathieu Conte, Thomas Gaillard, Floriane Denou, Mathieu Kerymel, Marion Guyon, Anthéa Loiez, Stéphanie Lebreton, Ferhat Meziani, Hayat Allam, Samir Chenaf, Hassène Rahmani, Sarah Heenen, Christine Kummerlen, Xavier Delabranche, Alexandra Boivin, Raphaël Clere-Jehl, Yannick Rabouël, Julien Pottecher, Sophie Bayer, Catherine Metzger, Stéphane Hecketsweiler, Pierre Olivier Ludes, Hortense Besancenot, Nadia Dhif, Guy Freys, Jean-Marc Lessinger, Anne Launoy, Aude Ruimy, Alain Meyer, M. Szozot, Alexandre Mebazaa, Nicolas Deye, Etienne Gayat, Marie-Céline Fournier, Sarra Abroug, Badr Louadah, Elodie Feliot, Sebastian Voicu, Isabelle Malissin, Bruno Megarbane, Philippe Manivet, Gardianot Victori, Da Silva Kelly, Béatrice La Foucher, Valérie Pierre, Lamia Kerdjana, Thomas Beeken, Antoine Goury, Pierre Garcon, Samuel Gaugain, Benjamin Glenn Chousterman, Benjamin Huot, Romain Barthelemy, Benjamin Soyer, Laurent Jacob, Matthieu Legrand, Marie-Céline Fournier, Francine Bonnet, Chloé Legall, Haikel Oueslati, Alexandru Cupaciu, Philippe Manivet, Badr Louadah, Romain Sonneville, Sophie Letrou, Lila Bouadma, Bruno Mourvillier, Véronique Deiler, Eric Magalhaes, Mathilde Neuville, Jean-François Timsit, Aguila Radjou, Stéphane Gaudry, Emeline Dubief, Jonathan Messika, Béatrice La Combe, Damien Roux, Guillaume Berquier, Mohamed Laissi, Jean-Damien Ricard, Jean-Michel Constantin, Sebastien Perbet, Julie Delmas, Julien Pascal, Sophie Cayot, Renaud Guerin, Matthieu Jabaudon, Laurence Roszyk, Christine Rolhion, Justine Bourdier, Mathilde Lematte, Charlène Gouhier, Camille Verlhac, Thomas Godet, Sophiano Radji, Elodie Caumon, Sandrine Thibault, Nikolaus Marx, Tobias Schuerholz, Jessica Pezechk, Florian Feld, Christian Brülls, Thorben Beeker, Tim-Philipp Simon, Robert Deisz, Achim Schindler, Bianca Meier, Thorsten Janisch, Andreas Hohn, Dirk Schedler, Wolfgang Wetsch, Daniel Schröder, Andreas Meier-Hellmann, Alexander Lucht, Robert Henker, Magdalena Römmer, Torsten Meinig, Kai D. Zacharowski, Patrick Meybohm, Simone Lindau, Haitham Mutlak, Stefan Kluge, Grit Ringeis, Birgit Füllekrug, Brigitte Singer, Axel Nierhaus, Katrin Bangert, Geraldine de Heer, Daniel Frings, Valentin Fuhrmann, Jakob Müller, Jörg Schreiber, Barbara Sensen, Stephanie Siedler, Annekatrin Siewecke, Gerold Söffker, Dominic Wichmann, Mélanie Kerinn, Ulrich Jaschinski, Ilse Kreuser, Marlene Zanquila, Andreas Kortgen, Frank Bloos, Falk Gonnert, Daniel Thomas-Rüddel, Anja Haucke, Steffi Kolanos, Karina Knuhr Kohlberg, Petra Bloos, Katrin Schwope, Salvatore Di Somma, Marino Rossella, Veronica Russo, Santarelli Simona, Christopher Bartoli, Sylvia Navarin, Cristina Bongiovanni, Michela Orru, Daniela Quatrocchi, Giada Zoccoli, Antonella Varchetta, Massimo Antonelli, Gennaro de Pascale, Maria Sole Vallecoccia, Salvatore Lucio Cutuli, Valentina Digravio, Daniela Quattrochi, Sonia D’Arrigo, Filippo Elvino Leone, Bert Beishuizen, Martin Rinket, Natalie Border, Mariska Bos-Burgmeijer, Astrid Braad, S. Papendorp, Alexander Cornet, J. Vermeijden, Ronald J. Trof, Peter Pickkers, Marieke van de A, Helen Van Wezel, Leo Heunks, Natalie Border, Chantal Luijten-Arts, Astrid Hoedemaekers, Hans van der Hoeven, Noortje Roovers, Pleun Hemelaar

**Affiliations:** 1 Department of Anesthesiology and Critical Care, Hôpital Lariboisière, DMU Parabol, APHP.Nord, Paris, France; 2 Inserm U942 MASCOT, Université de Paris, Paris, France; 3 Sphingotec GmbH, Hennigsdorf, Germany

To the editor:

Sepsis mortality decreased over the last decades, although it remains dramatically high [1]. The implementation of guidelines such as the Surviving Sepsis Campaign (SSC) contributed to these progresses. SSC recommends to guide resuscitation on normalization of lactate levels [2]. Guiding resuscitation on lactate reduction is highly debated [3]. Anyway, normalization of lactate is associated with improved outcome [4]. We have recently shown that plasma levels of bio-adrenomedullin (bio-ADM), a peptide regulating vascular integrity and endothelial function, were associated with patient outcome during sepsis [5]. Interestingly, we observed that patients with elevated bio-ADM levels at admission and with low bio-ADM levels 2 days later had similar outcome to patients with persistently low bio-ADM levels. We therefore aimed to evaluate the added value of bio-ADM to lactate measurement in the AdrenOSS-1 cohort.

The AdrenOSS-1 study is a prospective observational study conducted in 24 centers within 5 European countries and included 583 septic patients from June 2015 to May 2016 [5]. The primary endpoint was 28-day mortality. We evaluated the relationship between the association of initial evolution of lactate plasma levels and bio-ADM level at 24 h and outcome in patients for whom both markers were available at admission and 1 day later (“24 h”). As described previously, bio-ADM levels below or above 70 pg/mL were considered respectively as low and high [5].

In patients with high lactate levels (> 2 mmol/L) at admission (*n* = 328) (Table 1), lactate normalization (< 2 mmol/L) at 24 h was associated with better outcome than in patients with persistently high lactate at 24 h (28-day mortality 15.9% vs 41.9% respectively, HR 3.3 [2.0–5.3], *p* < 0.001) (Fig. 1).

**Table 1:** Clinical characteristics of septic patients admitted with a lactate level > 2 mmol/L and alive at 24 h (*n* = 269)

Patient characteristics	All	24 h lactate < 2 mmol/L and bio-ADM < 70 pg/mL	24 h lactate < 2 mmol/L and bio-ADM > 70 pg/mL	24 h lactate > 2 mmol/L and bio-ADM < 70 pg/mL	24 h lactate > 2 mmol/L and bio-ADM > 70 pg/mL	*p* value	Number of patients (if not indicated *n* = 269)
Number of patients (*n*, %)	269 (100)	75 (27.9)	70 (26.0)	28 (10.4)	96 (35.7)		
bio-ADM at admission (pg/ml)	113.7 [59.3–206.4]	46.7 [33.1–63.0]	137.3 [103.2–217.8]	61.5 [36.3–84.3]	192.4 [129.0–355.6]	< 0.0001	
Lactate at admission (mmol/l)	3.6 [2.6–5.5]	2.8 [2.3–3.5]	3.3 [2.5–4.5]	3.5 [2.7–4.6]	5.4 [3.5–8.8]	< 0.0001	
Age (years)	65.7 [54.7–75.6]	64.0 [54.4–71.8]	65.7 [58.5–74.3]	67.6 [56.8–76.9]	67.8 [54.6–77.4]	0.4697	
Male sex (*n*, %)	171 (63.6)	52 (69.3)	45 (64.3)	18 (64.3)	56 (58.3)	0.5253	
Body mass index (kg/m^2^)	26.1 [23.1–30.8]	26.1 [23.9–29.4]	25.1 [20.5–30.4]	26.4 [22.9–31.3]	27.3 [23.6–31.8]	0.3834	*n* = 232
Septic shock at admission (*n*, %)	172 (63.9)	34 (45.3)	46 (65.7)	15 (53.6)	77 (80.2)	0.0001	
Type of ICU admission						0.1378	
Medical (*n*, %)	198 (73.6)	62 (82.7)	49 (70.0)	24 (85.7)	63 (65.6)		
Surgical—emergency procedure (*n*, %)	60 (22.3)	10 (13.3)	18 (25.7)	4 (14.3)	28 (29.2)		
Surgical—elective procedure (*n*, %)	11 (4.1)	3 (4.0)	3 (4.3)	0 (0.0)	5 (5.2)		
Origin of sepsis						0.0156	
Lung (*n*, %)	87 (32.3)	28 (37.3)	16 (22.9)	15 (53.6)	28 (29.2)		
Bloodstream (*n*, %)	35 (13)	14 (18.7)	8 (11.4)	4 (14.3)	9 (9.4)		
Urinary tract (*n*, %)	46 (17.1)	4 (5.3)	15 (21.4)	4 (14.3)	23 (24)		
Catheter (*n*, %)	15 (5.6)	4 (5.3)	3 (4.3)	3 (10.7)	5 (5.2)		
Peritonitis (*n*, %)	16 (5.9)	6 (8.0)	3 (4.3)	0 (0.0)	7 (7.3)		
Endocarditis (*n*, %)	14 (5.2)	4 (5.3)	4 (5.7)	1 (3.6)	5 (5.2)		
Bile duct infection (*n*, %)	4 (1.5)	0 (0.0)	2 (2.9)	0 (0.0)	2 (2.1)		
CNS (*n*, %)	1 (0.4)	1 (1.3)	0 (0.0)	0 (0.0)	0 (0.0)		
Skin and soft tissue (*n*, %)	4 (1.5)	4 (5.3)	0 (0.0)	0 (0.0)	0 (0.0)		
Gynecologic (*n*, %)	1 (0.4)	0 (0.0)	0 (0.0)	0 (0.0)	1 (1.0)		
Other (*n*, %)	46 (17.1)	10 (13.3)	19 (27.1)	1 (3.6)	16 (16.7)		
Medical history							
Any cardiac comorbidity (*n*, %)	184 (68.4)	43 (57.3)	49 (70)	18 (64.3)	74 (77.1)	0.0481	
Chronic heart failure (*n*, %)	29 (10.9)	6 (8.0)	5 (7.2)	3 (11.1)	15 (15.8)	0.2684	
Hypertension (*n*, %)	143 (53.8)	33 (44.0)	38 (55.1)	14 (50.0)	58 (61.7)	0.1407	
Diabetes mellitus (*n*, %)	76 (28.4)	21 (28.0)	19 (27.5)	3 (10.7)	33 (34.4)	0.1102	
Any noncardiac comorbidity (*n*, %)	198 (73.6)	51 (68.0)	55 (78.6)	21 (75.0)	71 (74.0)	0.5447	
Chronic renal disease (*n*, %)	31 (11.7)	6 (8.1)	10 (14.5)	2 (7.1)	13 (13.7)	0.4978	
Active/recent malignant tumors (*n*, %)	60 (22.5)	10 (13.3)	19 (27.9)	7 (25.0)	24 (25.0)	0.1565	
Smoking (active) (*n*, %)	57 (21.8)	17 (23.0)	15 (22.1)	5 (19.2)	20 (21.5)	0.9827	
COPD (*n*, %)	35 (13.1)	9 (12.0)	12 (17.4)	5 (17.9)	9 (9.5)	0.4156	
Any chronic medication (*n*, %)	176 (65.4)	42 (56.0)	53 (75.7)	16 (57.1)	65 (67.7)	0.0632	
Immunosuppressive therapy (*n*, %)	26 (9.7)	5 (6.7)	5 (7.1)	3 (10.7)	13 (13.5)	0.3963	
Physiological values at admission							
Temperature (°C)	37.2 [36.3–38.3]	37.2 [36.4–38.3]	37.2 [36.4–38.2]	36.9 [35.8–37.7]	37.2 [36.3–38.4]	0.6926	
Mean blood pressure (mmHg)	73 [62–92]	82 [68.5–99]	70.5 [60–84]	77.5 [58–94.2]	69 [58.5–86]	0.0009	*n* = 266
Heart rate (beats/min)	108 [96–122]	110 [93–123.5]	107 [95.2–118.7]	106 [97.7–115]	112.5 [97.7–130.2]	0.2976	
Central venous pressure (mmHg)	8 [5–12]	8 [5–13]	7 [3–11]	8 [7–8]	9 [6–12]	0.3535	*n* = 75
Glasgow Coma Scale score (points)	15 [13–15]	15 [14–15]	15 [14–15]	14 [13–15]	15 [13–15]	0.4721	*n* = 253
Fluid balance (mL)	2500 [1141–4716]	1930 [892–2626]	2156 [1375–3939]	2820 [1292–4323]	3657 [1426–5750]	0.0002	*n* = 235
Urine output for 24 h (mL)	1000 [354–1867]	1350 [941–2667]	675 [301–1619]	1562.5 [951–2220]	600 [177–1480]	< 0.0001	*n* = 248
PaO_2_/FiO_2_	220 [131–330]	254 [155–362]	231 [145–321]	211 [96–330]	190 [115–314]	0.1637	*n* = 244
Laboratory values at admission							
Arterial pH	7.36 [7.27–7.42]	7.41 [7.34–7.45]	7.37 [7.26–7.42]	7.38 [7.31–7.44]	7.31 [7.22–7.38]	< 0.0001	*n* = 261
Bilirubin (μmol/L)	12 [7–22]	13 [6.75–22.2]	11 [5.5–20.5]	12 [8–20.5]	12 [7–22]	0.7229	*n* = 259
Platelets (10^9^/L)	188 [116–265]	180 [128–261]	176 [110–284]	243 [135–336]	181 [110–245]	0.2770	*n* = 268
Creatinine (mg/dL)	1.5 [1.02–2.26]	1.13 [0.85–1.63]	1.79 [1.23–2.65]	1.03 [0.74–1.45]	1.72 [1.2–2.62]	< 0.0001	
Urea (mg/dL)	66 [41–109.91]	50.45 [36.04–78.34]	85.29 [53.6–118.77]	52 [33.48–77.27]	73.57 [46.7–120.84]	0.0001	
Hematocrit (%)	35 [30–39]	36 [30–39]	35 [30–40]	35 [31–37]	34 [29–40]	0.9579	*n* = 265
White blood cell count (per mm^3^)	11,690 [6037–18,142]	13,400 [8390–18,700]	11,115 [5497–16,500]	11,770 [7780–15,950]	10,780 [4200–17,722]	0.1827	*n* = 268
Troponin T, maximum at admission (ng/mL)	41.73 [18–219]	24 [14–50.5]	40.86 [19.5–126.75]	14 [13–47]	87.5 [27.82–329.25]	0.0535	*n* = 73
Troponin I, maximum at admission (ng/mL)	100 [29.9–323]	79 [19.25–327.23]	135 [37.02–233.68]	114.95 [22.48–230]	100 [31.9–312.95]	0.9752	*n* = 77
PCT, maximum at admission (ng/mL)	19.17 [6.33–79.32]	10.36 [4.35–37.93]	27.62 [7.75–60]	5.42 [2.24–11.21]	43.64 [9.6–103.41]	0.0054	*n* = 144
PCT, central laboratory (ng/mL)	15.34 [5.37–48.43]	8.21 [2.4–18.21]	22.55 [9.68–53.25]	7.12 [2.04–20.73]	29.22 [8.73–64.8]	< 0.0001	*n* = 269
BNP, maximum at admission (pg/mL)	376.2 [159–1132]	376.2 [169.5–1011]	356.1 [228–540.2]	219 [143.7–324]	757 [141.7–1619.5]	0.4335	*n* = 49
NT-proBNP, maximum at admission (pg/mL)	5119 [1620–17,118]	1847 [621–6709]	3873 [2594–23,052]	792 [249–3074]	7097 [4884–24,340]	0.0135	*n* = 54
Organ support at admission							
Mechanical ventilation						0.0008	
Invasive (*n*, %)	125 (46.5)	24 (32.0)	29 (41.4)	12 (42.9)	60 (62.5)		
Noninvasive (*n*, %)	49 (18.2)	16 (21.3)	9 (12.9)	7 (25.0)	17 (17.7)		
None (*n*, %)	95 (35.3)	35 (46.7)	32 (45.7)	9 (32.1)	19 (19.8)		
Renal replacement therapy (*n*, %)	28 (10.4)	1 (1.3)	7 (10.0)	3 (10.7)	17 (17.7)	0.0070	
Vasopressors/inotropes at admission (*n*, %)	192 (71.4)	41 (54.7)	51 (72.9)	18 (64.3)	82 (85.4)	0.0001	
ICU scoring systems							
SOFA (points)	8 [6–11]	6 [4–9]	8 [7–11]	8 [5–9]	10 [7–11.5]	< 0.0001	*n* = 240
APACHE II (points)	17 [13–22]	15 [10–18]	17 [12.2–21]	18.5 [13.7–23]	19 [15–23.2]	< 0.0001	
ICU length of stay (days)	6 [3–11]	5 [3–7.5]	7 [4–13]	5.5 [2.7–9.5]	7 [3–16.2]	0.0170	
Mortality							
28-day, deaths (*n*, %)	75 (27.9)	5 (6.7)	18 (25.7)	4 (14.3)	48 (50.0)	< 0.0001	
90-day, deaths (*n*, %)	93 (34.6)	10 (13.3)	22 (31.4)	6 (21.4)	55 (57.3)	< 0.0001	

Data are presented as median [IQR] or *n* (%)

**Fig. 1:**
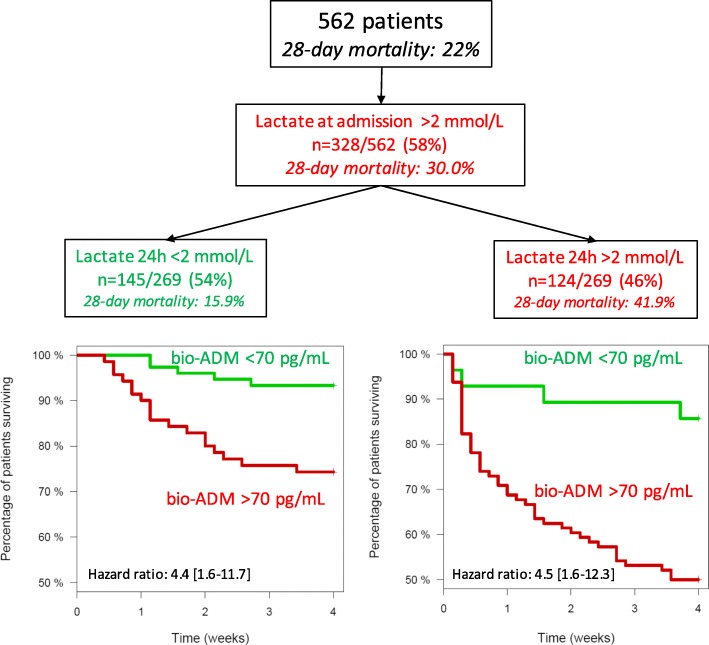
Impact of 24 h lactate and bio-ADM values in patients with elevated lactate level at admission. The green curve in the left KM-plot illustrates data from 75 patients with 5 events, the red curve 70 patients with 18 events. The green curve in the right KM-plot illustrates data from 28 patients with 4 events, the red curve 96 patients with 48 events. Of note, differences in numbers between admission (*n* = 328) and 24 h (*n* = 269) is related to initial mortality

Interestingly, among patients with decreasing lactate, high and low bio-ADM levels at 24 h identified patients with substantially different outcomes (28-day mortality 7% vs 26% for low vs high bio-ADM respectively, HR 4.4 [1.6–11.7], *p* < 0.005) (Fig. 1). High and low bio-ADM levels at 24 h also differentiated outcome of patients with persistently elevated lactate (HR 4.5 [1.6–12.3], *p* < 0.005).

In patients with low initial lactate (*n* = 234 admitted and *n* = 171 alive at 24 h), overall 28-day mortality was 11.2%, neither lactate nor bio-ADM added prognostic value.

For all analyses, similar results were obtained, when missing 24 h data were replaced by the last available values.

Accordingly, our data suggest that measurement of bio-ADM in addition to lactate may help physicians to refine risk stratification and therefore to guide resuscitation during sepsis.

## Data Availability

AM had full access to all data in the study and take responsibility for the integrity of the data and the accuracy of the data analysis.
